# Supplementary material for: A mechanical-coupling mechanism in OSCA/TMEM63 channel mechanosensitivity
Source: Nat Commun. 2023 Jul 4;14:3943. doi: 10.1038/s41467-023-39688-8 (PMC10319725; doi:10.1038/s41467-023-39688-8)
Supplement: Supplementary file 1 — Supplementary Information [file 41467_2023_39688_MOESM1_ESM.pdf]

Supplementary Information for

A mechanical-coupling mechanism in OSCA/TMEM63 channel mechanosensitivity

Mingfeng Zhang[1,2,#*], Yuanyue Shan[1,2,#], Charles D Cox[3,4*], Duanqing Pei[2*]

[1] Fudan University, Shanghai, 200433, China.

[2] Laboratory of Cell Fate Control, School of Life Sciences, Westlake University, Hangzhou, 310000, China.

[3] Victor Chang Cardiac Research Institute, Sydney, 2010, Australia.

[4] School of Biomedical Sciences, Faculty of Medicine & Health, UNSW Sydney, Kensington, New South Wales, 2052, Australia.

#These authors contributed equally

*Corresponding to : peiduanqing@westlake.edu.cn.or zhangmingfeng@westlake.edu.cn or c.cox@victorchang.edu.au

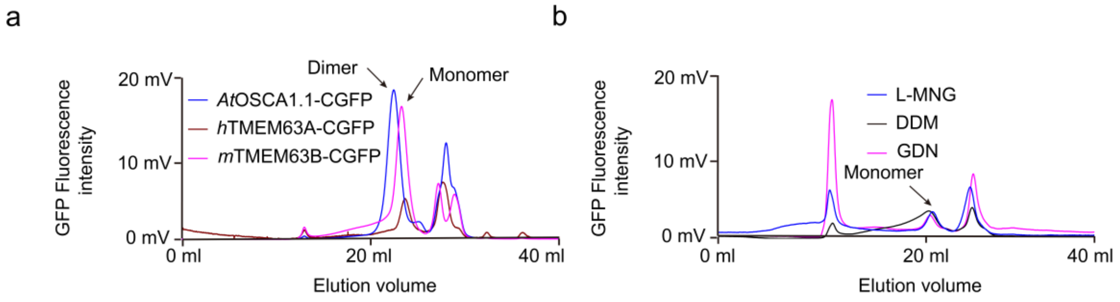

Supplementary Fig. 1 *m*TMEM63B and *h*TMEM63A are monomeric in a detergent environment. a, Representative fluorescence-detection size-exclusion chromatography (FSEC) traces of *m*TMEM63B (pink) and *h*TMEM63A (brown) are compared to that of the *AtOSCA*1.1 (blue). The position of the *AtOSCA*1.1 dimer peak, *m*TMEM63B and *h*TMEM63A monomer peaks are denoted by black arrows. **b,** Representative fluorescence-detection size-exclusion chromatography (FSEC) traces of different detergent extracted *h*TMEM63A, all of which suggested monomeric states of *h*TMEM63A (L-MNG, DDM and GDN).

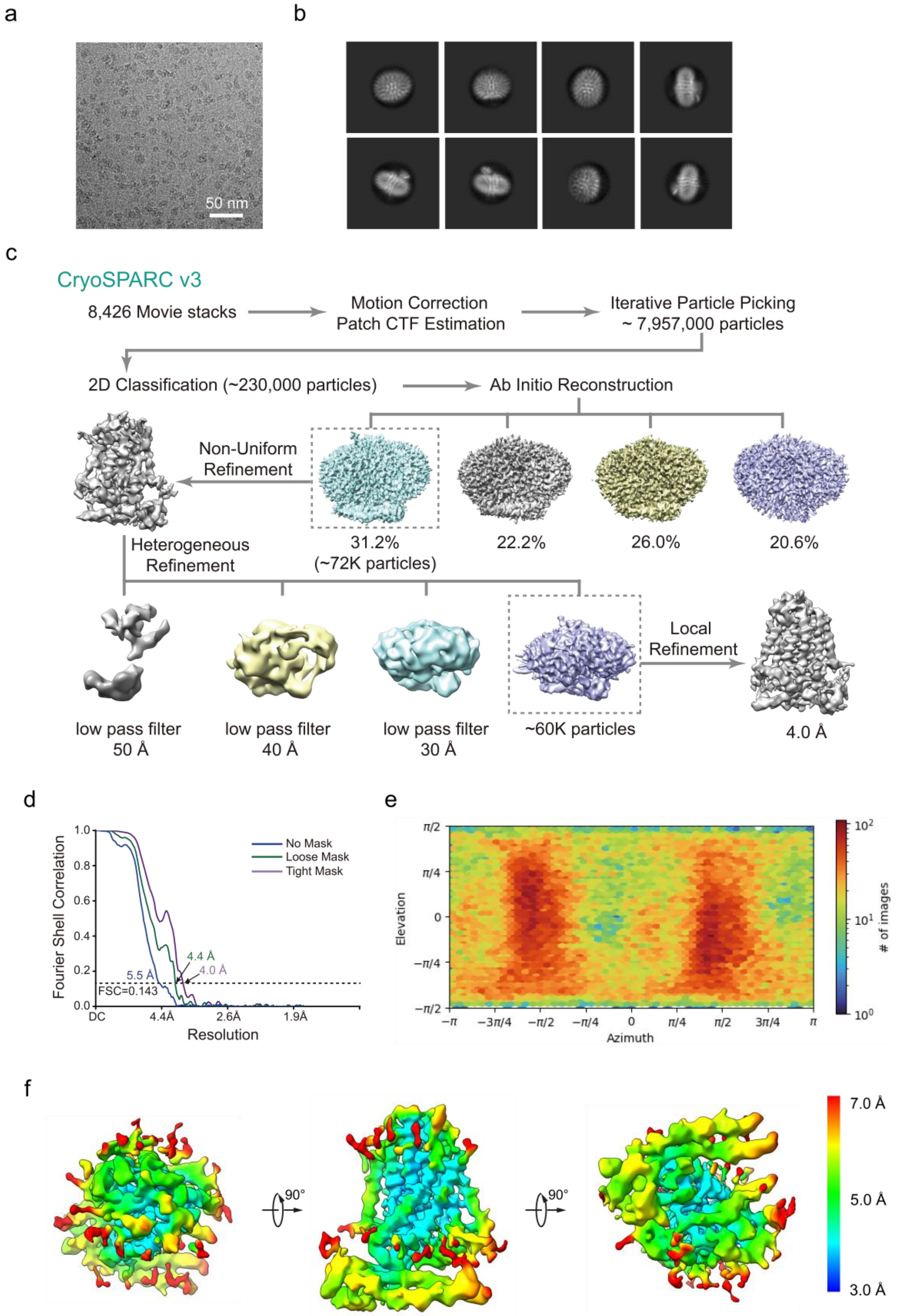

Supplementary Fig. 2 Cryo-EM data processing procedure of *m*TMEM63B. a, Representative raw micrograph of *m*TMEM63B in a detergent environment from 8,426 collected micrographs. **b**, Representative 2D class averages of the cryo-EM particles of *m*TMEM63B in a detergent environment. **c**, Flowchart of the image processing procedure

for *m*TMEM63B in a detergent environment. **d**, Gold-standard Fourier shell correlation (FSC) curves of the final refined maps for unmasked (blue line), loose masked (green line) and tight masked (purple line). Resolution estimation (5.5 Å for the unmasked map, 4.4 Å for the loose masked map and 4.0 Å for the tight masked map) is based on the criterion of an FSC cutoff of 0.143. **e**, Angular distribution histogram of the final *m*TMEM63B reconstruction. This is a standard output from cryoSPARC. **f**, Local resolution of *m*TMEM63B in the top view (left), side view (middle) and bottom view (right) is shown.

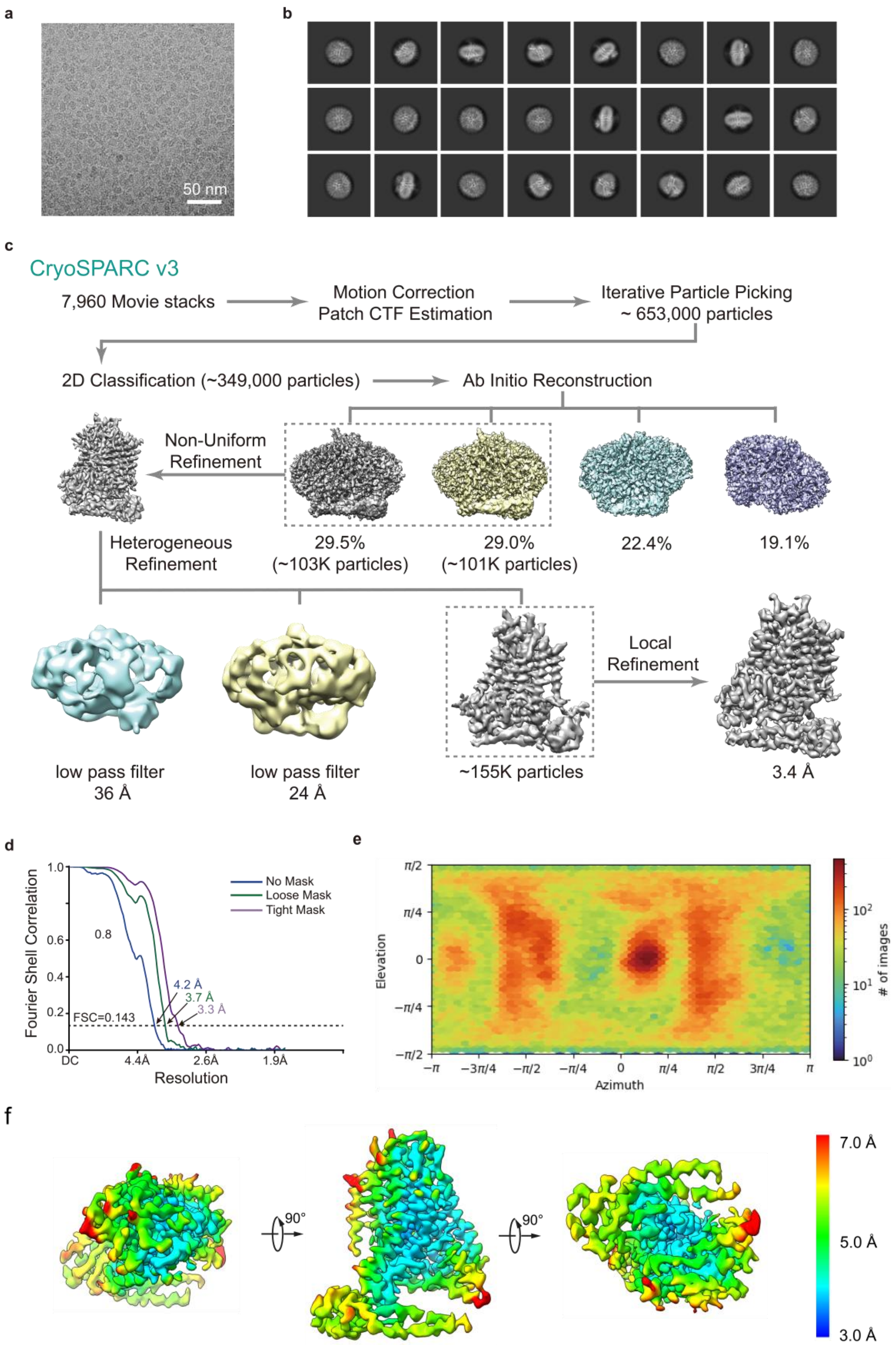

Supplementary Fig. 3 Cryo-EM data processing procedure of *h*TMEM63A. a, Representative raw micrograph of *h*TMEM63A from 7,960 collected micrographs. **b**, Representative 2D class averages of the cryo-EM particles of *h*TMEM63A. **c**, Flowchart of

the image processing procedure for *h*TMEM63A. **d**, Gold-standard Fourier shell correlation (FSC) curves of the final refined maps for unmasked (blue line), loose masked (green line) and tight masked (purple line). Resolution estimation (4.2 Å for the unmasked map, 3.7 Å for the loose masked map and 3.3 Å for the tight masked map) is based on the criterion of an FSC cutoff of 0.143. **e**, Angular distribution histogram of the final *h*TMEM63A reconstruction. This is a standard output from cryoSPARC. **f**, Local resolution of *h*TMEM63A in top view (left), side view (middle) and bottom view (right) is shown.

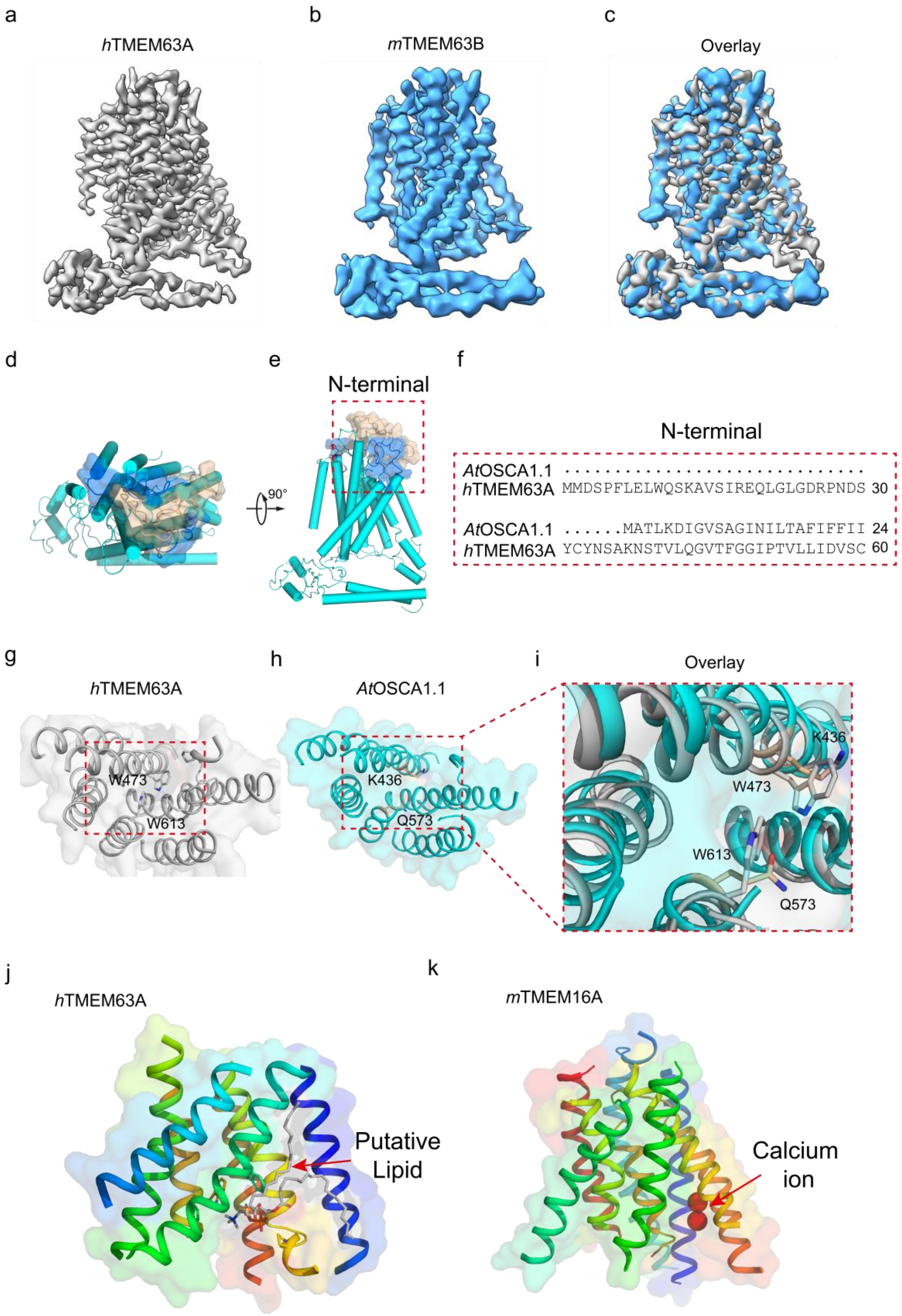

Supplementary Fig. 4 Structure comparison among *AtOSCA*1.1, *h*TMEM63A and *m* TMEM63B. a-c, Cryo-EM density maps of *h*TMEM63A in grey (a) and *m*TMEM63B in blue (b) and superimposition of them (c). **d-e**, Cartoon representation of *h*TMEM63A viewed from top (d) and side (e). The extra N-terminal portion of *h*TMEM63A when compared to

*At*OSCA1.1 is shown in beige and the conserved portion shown in blue. **f**, Sequence alignment of N-terminal residues of *AtOSCA*1.1 and *h*TMEM63A. *h*TMEM63A has around 36 additional residues comparing to *At*OSCA1.1. **g-h,** Cartoon and transparent surface representation of the putative lipid binding site of *h*TMEM63A (g) and *At*OSCA1.1 (h). The residues W473 and W613 interacting with lipid and the corresponding residues K436 and Q573 that would interact with the lipid in *AtOSCA*1.1 are labelled. **i**, Enlarged overlay of the lipid binding site in *h*TMEM63A and *AtOSCA*1.1. **j-k,** The pore region plugged by a lipid in *h*TMEM63A (j) and the corresponding region and calcium ion in *m*TMEM16A (k).

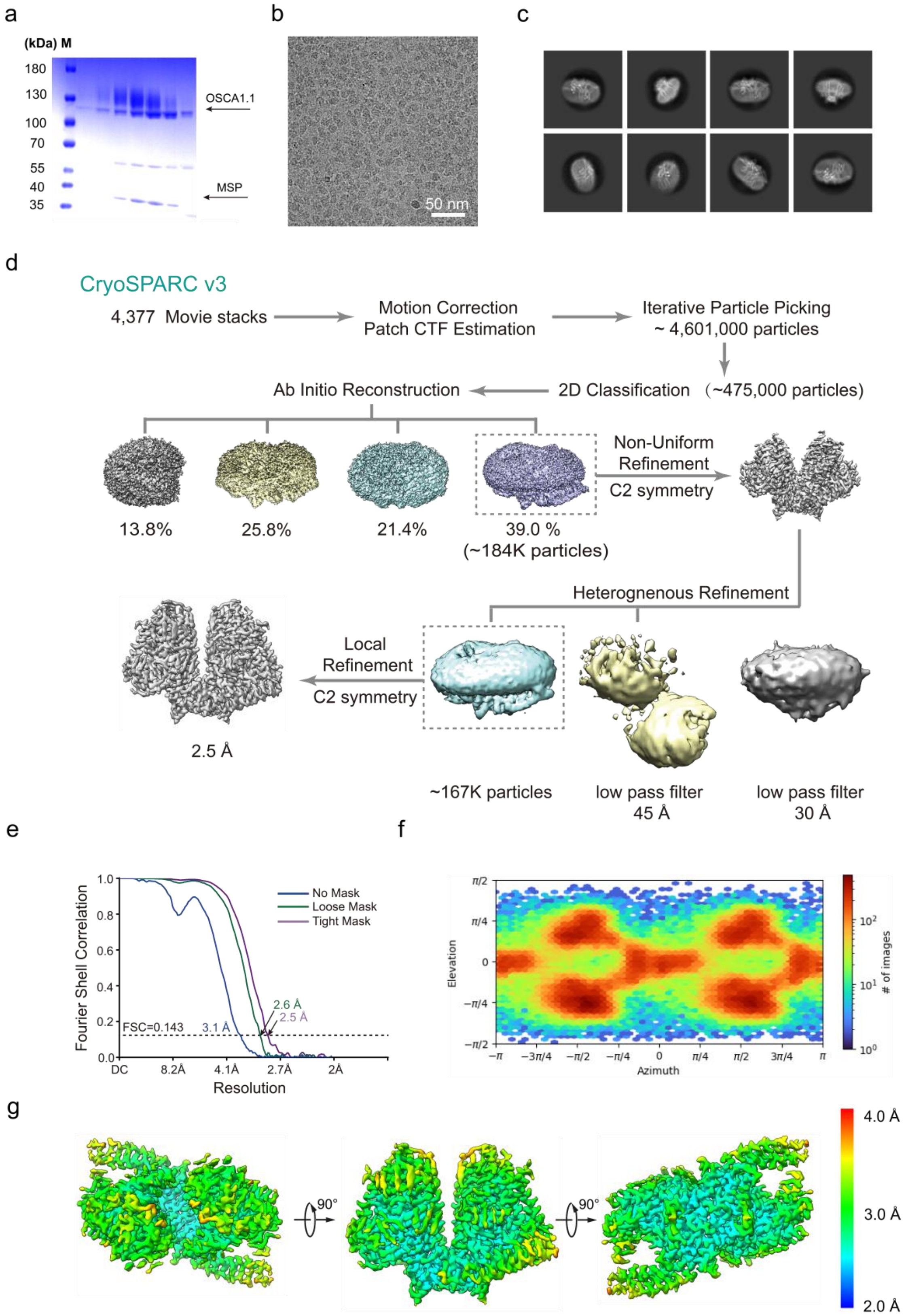

Supplementary Fig. 5 Cryo-EM data processing procedure of *AtOSCA*1.1 in nanodisc. a, *AtOSCA*1.1 protein samples of the size-exclusion chromatography (SEC) fractions were subjected to SDS-PAGE and Coomassie blue staining. Bands of *AtOSCA*1.1 and MSPE3D1 are denoted by black arrows. SDS-PAGE and Coomassie blue

staining was performed only once, as the image quality was high enough to be convincing. **b,** Representative raw micrograph of *AtOSCA*1.1 in nanodisc from 4,377 collected micrographs. **c**, Representative 2D class averages of the cryo-EM particles of *AtOSCA*1.1 in nanodisc. **d**, Flowchart of the image processing procedure for *AtOSCA*1.1 in nanodisc. **e**, Gold-standard Fourier shell correlation (FSC) curves of the final refined maps for unmasked (blue line), loose masked (green line) and tight masked (purple line). Resolution estimation (3.1 Å for the unmasked map, 2.6 Å for the loose masked map and 2.5 Å for the tight masked map) is based on the criterion of an FSC cutoff of 0.143. **f**, Angular distribution histogram of the final *AtOSCA*1.1 in nanodisc reconstruction. This is a standard output from cryoSPARC. **g**, Local resolution of *AtOSCA*1.1 in nanodisc in top view (left), side view (middle) and bottom view (right) is shown.

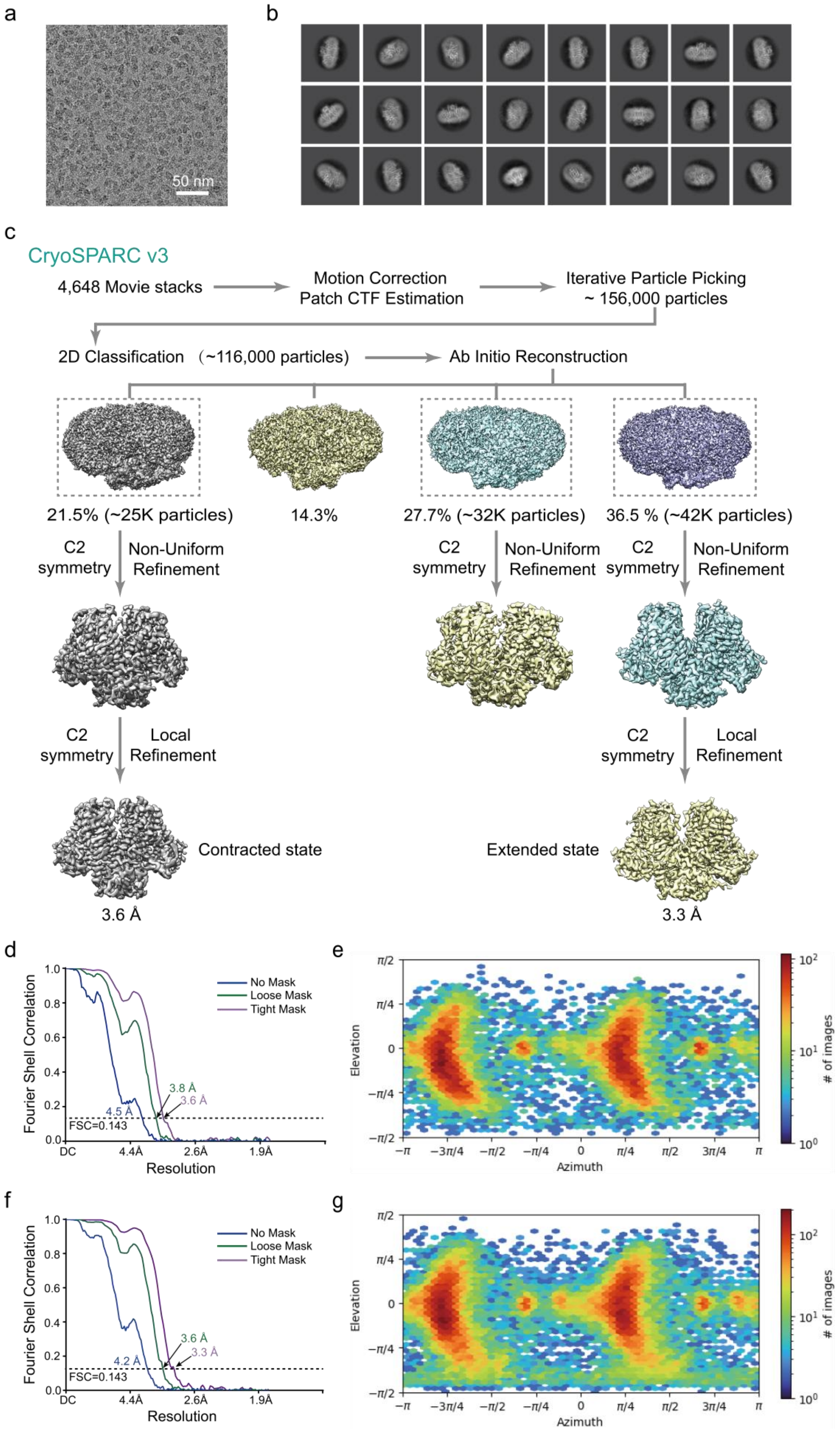
a
50 nm
b
c
CryoSPARC v3
4,648 Movie stacks
Motion Correction
Patch CTF Estimation
Iterative Particle Picking
~ 156,000 particles
2D Classification (~116,000 particles)
Ab Initio Reconstruction
21.5% (~25K particles)
14.3%
27.7% (~32K particles)
36.5 % (~42K particles)
C2 symmetry
Non-Uniform Refinement
C2 symmetry
Local Refinement
Contracted state
3.6 Å
Extended state
3.3 Å
d
Fourier Shell Correlation
No Mask
Loose Mask
Tight Mask
3.8 Å
3.6 Å
4.5 Å
FSC=0.143
DC
4.4Å
2.6Å
1.9Å
Resolution
e
Elevation
Azimuth
# of images
f
Fourier Shell Correlation
No Mask
Loose Mask
Tight Mask
3.6 Å
3.3 Å
4.2 Å
FSC=0.143
DC
4.4Å
2.6Å
1.9Å
Resolution
g
Elevation
Azimuth
# of images

Supplementary Fig. 6 Cryo-EM data processing procedure of *AtOSCA*3.1 in detergent. **a**, Representative raw micrograph of *AtOSCA*3.1 in detergent from 4,648 collected micrographs. **b**, Representative 2D class averages of the cryo-EM particles of *AtOSCA*3.1 in detergent. **c**, Flowchart of the image processing procedure for *AtOSCA*3.1 in detergent. **d**, Gold-standard Fourier shell correlation (FSC) curves of the final refined maps of the contracted *AtOSCA*3.1 for unmasked (blue line), loose masked (green line) and tight masked (purple line). Resolution estimation (4.5 Å for the unmasked map, 3.8 Å for the loose masked map and 3.6 Å for the tight masked map) is based on the criterion of an FSC cutoff of 0.143. **e**, Angular distribution histogram of the final *AtOSCA*3.1 in detergent reconstruction. This is a standard output from cryoSPARC. **f**, Gold-standard Fourier shell correlation (FSC) curves of the final refined maps of the extended *AtOSCA*3.1 for unmasked (blue line), loose masked (green line) and tight masked (purple line). Resolution estimations (4.2 Å for the unmasked map, 3.6 Å for the loose masked map and 3.3 Å for the tight masked map) based on the criterion of an FSC cutoff of 0.143. **g**, Local resolution of *AtOSCA*3.1 in nanodisc in top view (left), side view (middle) and bottom view (right) is shown.

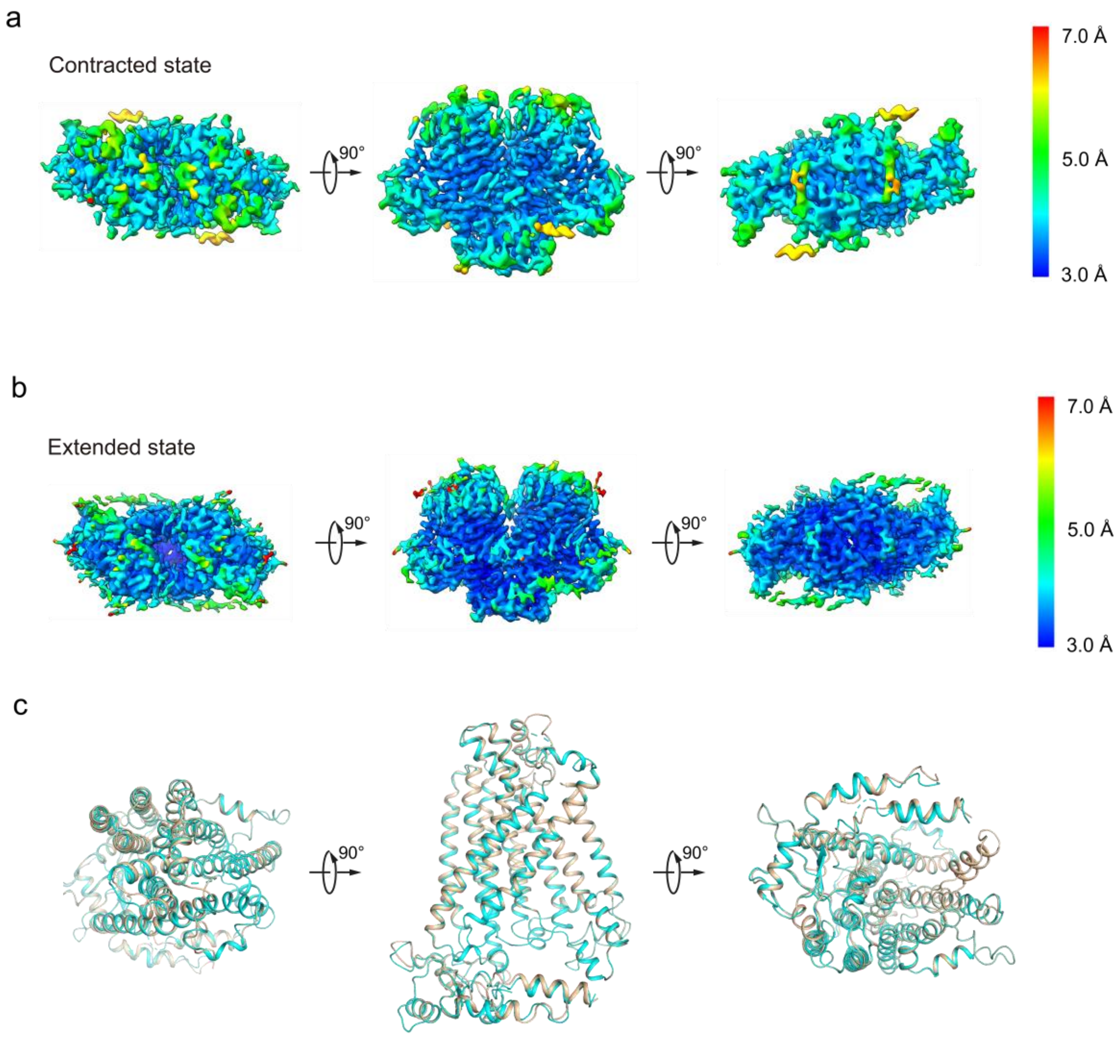

Supplementary Fig. 7 Local resolution of the contracted and extended state of *At*OSCA3.1 estimated by local resolution estimation. a-b, Local resolution of the

contracted **(a)** and extended state **(b)** of *AtOSCA*3.1 in the top view (left), side view (middle) and bottom view (right) are shown. **c,** Structural comparison of a single subunit of the contracted and extended states of *At*OSCA3.1 in top view (left), side view (middle) and bottom view (right), the RMSD=0.121 Å. c, Superimposition of the contracted (beige) and extended states (blue) of *AtOSCA*3.1

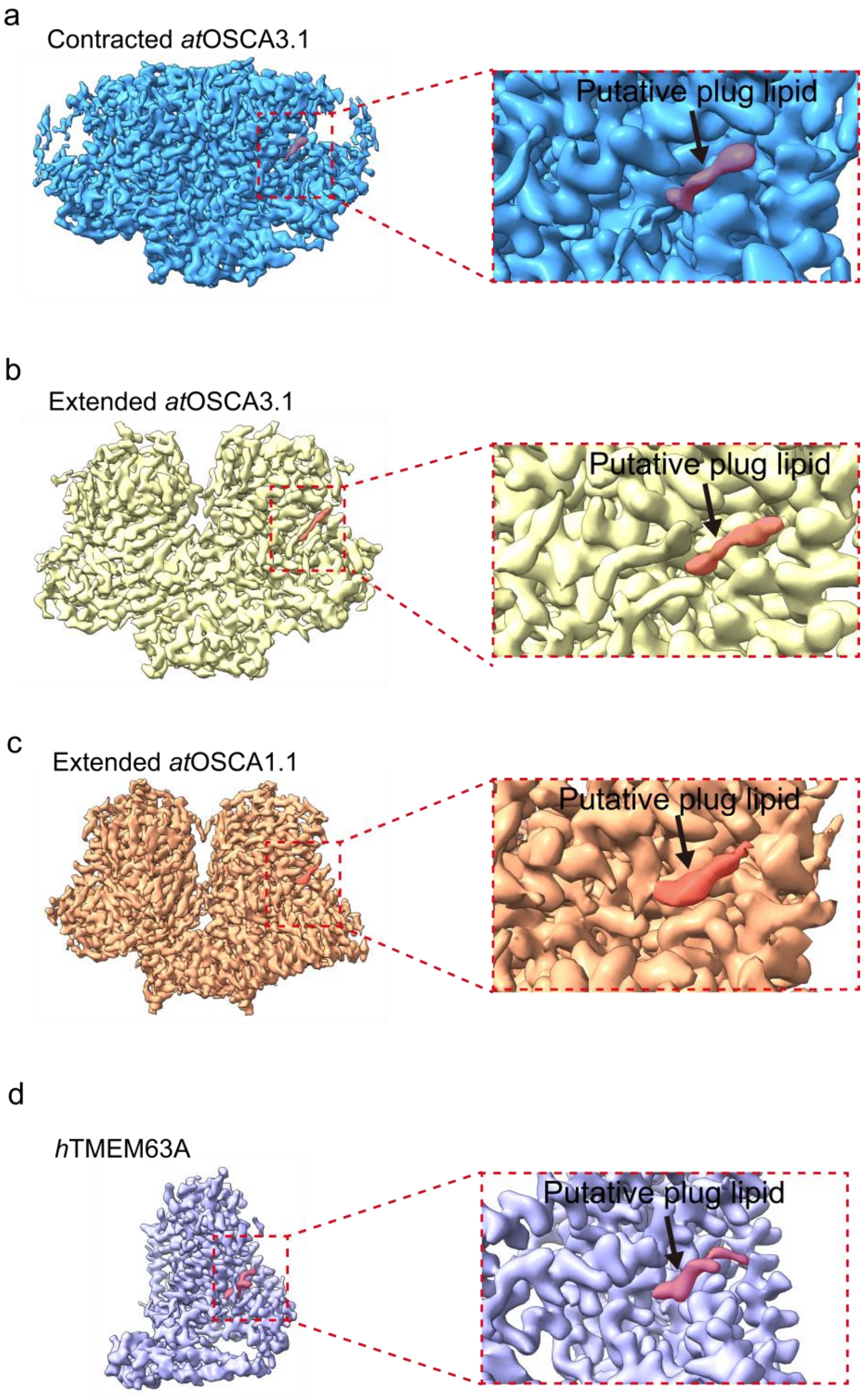

Supplementary Fig. 8 The putative lipid plug density for all OSCA/TMEM63 structures. a-d, The cryo-EM density map of contracted *AtOSCA*3.1(a), extended *AtOSCA*3.1(b), extended *AtOSCA*1.1(c) and *h*TMEM63A(d). The red box indicated the cytosolic pore region plugged by the putative plug-lipid. The plug-lipid is indicated by the

arrow in the zoom view of the cytosolic portion of the pore.

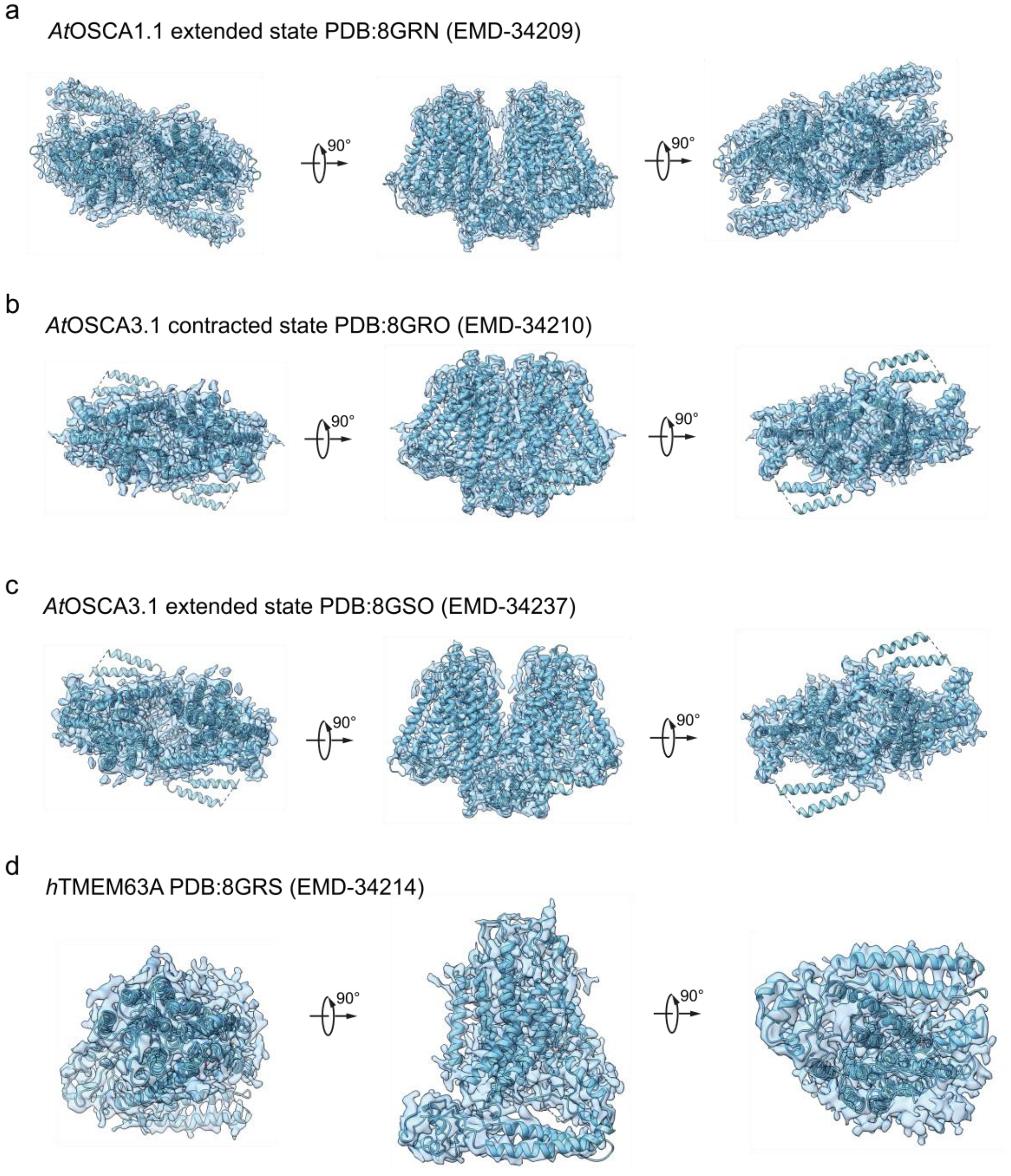

Supplementary Fig. 9 The model fit to cryo-EM map of all OSCA/TMEM63 structures. a-d, The model fit to cryo-EM density map of extended *AtOSCA*1.1(a), contracted *AtOSCA*3.1(b), extended *AtOSCA*3.1(c), and *h*TMEM63A(d) in the top view (left), side view (middle) and bottom view (right) are shown.

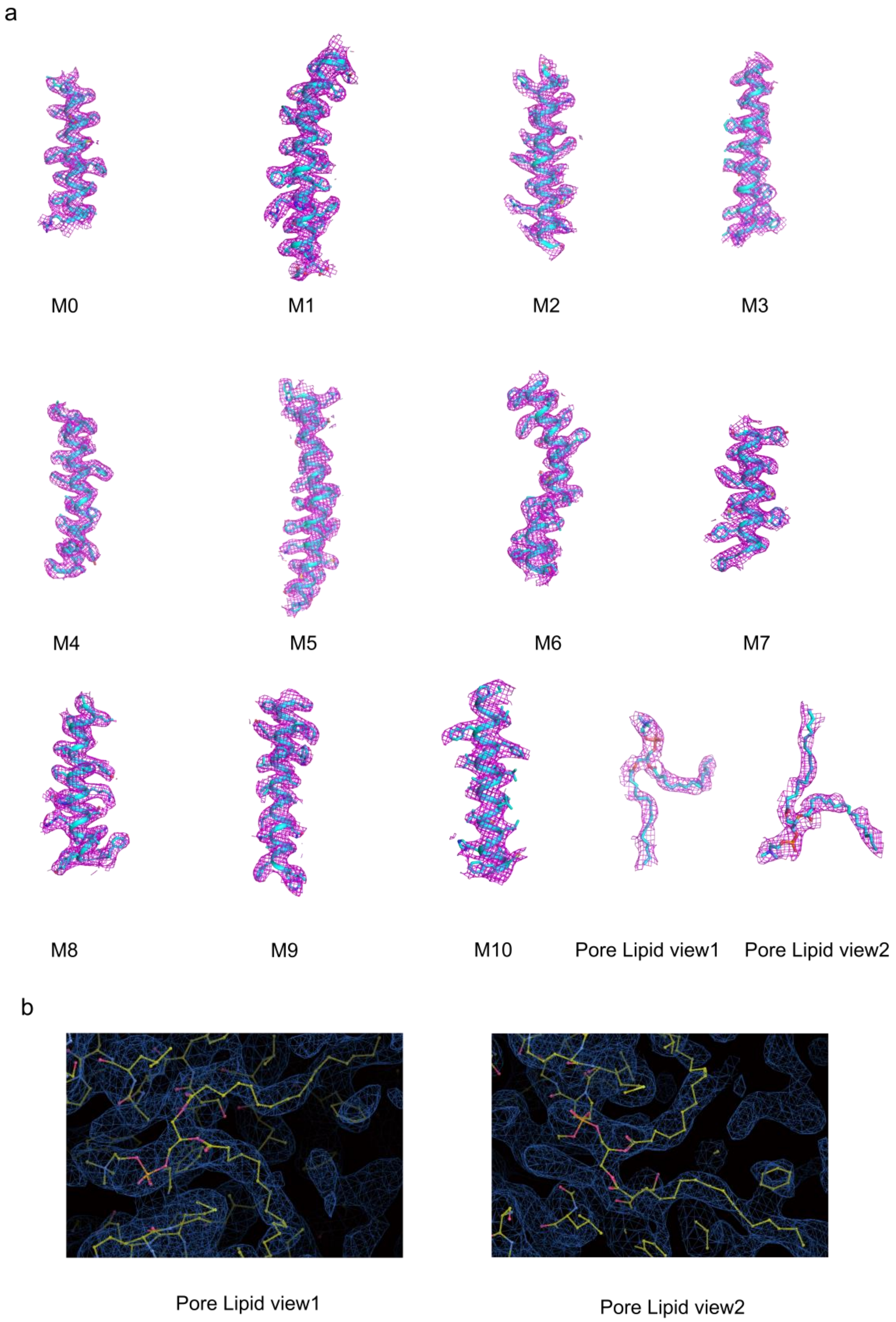

Supplementary Fig. 10 The EM density of *h*TMEM63A. a, The EM density of the transmembrane domain of *h*TMEM63A (M0-M10) and the pore lipid of two rotated views. **b,** The two rotated views of the pore lipid are shown in Coot software.

Supplementary Table 1. Statistics for data collection and structural refinement

	#1 AtOSCA1.1 extended state 8GRN (EMD-34209)	#2 AtOSCA3.1 contracted state 8GRO (EMD- 34210)	#3 AtOSCA3.1 extended state 8GSO (EMD- 34237)	#4 hTMEM63A 8GRS (EMD- 34214)
**Data collection and processing**				
Microscope	FEI Titan Krios	FEI Titan Krios	FEI Titan Krios	FEI Titan Krios
Magnification	105000	105000	105000	105000
Voltage (KV)	300	300	300	300
Detector	Gatan K3	Gatan K3	Gatan K3	Gatan K3
Electron exposure (e-/ Å2)	50	50	50	50
Defocus range (μm)	-1.5 to -2.0	-1.5 to -2.0	-1.5 to -2.0	-1.5 to -2.0
Pixel size (Å)	0.847	0.834	0.834	0.82
Symmetry imposed	C2	C2	C2	C1
Initial particle images (no.)	2043870	115669	115669	349445
Final particle images (no.)	838335	24872	42184	155709
Map resolution (Å)	2.5	3.55	3.29	3.35
FSC threshold	0.143	0.143	0.143	0.143
**Refinement**				
Initial model used (PDB code)	6JPF	6JPF	6JPF	6JPF
Model resolution (Å)	2.5	3.5	3.3	3.3
FSC threshold	0.143	0.143	0.143	0.143
Map sharpening B factor (Å2)	87.5	96.7	102.2	151.4
**Model composition**				
Non-hydrogen atoms	11540	10628	10553	4846
Protein residues	1432	1322	1312	626
Water				
Ions				
Detergents				
**B factors**				
Protein	38.77	147.95	73.13	96.83
Water				
**R.m.s. deviations**				

Bond lengths (Å)	0.004	0.004	0.004	0.004
Bond angles (°)	1.077	0.917	0.89	0.88
**Validation**				
MolProbity score	2.34	2.27	2.16	2.28
Clashscore	14.02	15.34	12.55	13.72
Poor rotamers (%)	0	0	0	0
**Ramachandran plot**				
Favored (%)	83.68	88.74	89.85	86.36
Allowed (%)	13.94	9.97	9.23	12.66
Disallowed (%)	2.38	1.29	0.92	0.97

Additional information

The source data underlying Supplementary Fig. 5a is provided as a Source Data file.
