# Supplementary material for: A mechanical-coupling mechanism in OSCA/TMEM63 channel mechanosensitivity
Source: Nat Commun. 2023 Jul 4;14:3943. doi: 10.1038/s41467-023-39688-8 (PMC10319725; doi:10.1038/s41467-023-39688-8)
Supplement: Supplementary file 3 — Reporting Summary [file 41467_2023_39688_MOESM3_ESM.pdf]

Corresponding author(s): Duanqing Pei

Last updated by author(s): May 20, 2023

Reporting Summary

Nature Portfolio wishes to improve the reproducibility of the work that we publish. This form provides structure for consistency and transparency in reporting. For further information on Nature Portfolio policies, see our Editorial Policies and the Editorial Policy Checklist.

Statistics

For all statistical analyses, confirm that the following items are present in the figure legend, table legend, main text, or Methods section.

n/a	Confirmed	
☐	☒	The exact sample size (n) for each experimental group/condition, given as a discrete number and unit of measurement
☐	☒	A statement on whether measurements were taken from distinct samples or whether the same sample was measured repeatedly
☐	☒	The statistical test(s) used AND whether they are one- or two-sided<br>*Only common tests should be described solely by name; describe more complex techniques in the Methods section.*
☒	☐	A description of all covariates tested
☒	☐	A description of any assumptions or corrections, such as tests of normality and adjustment for multiple comparisons
☐	☒	A full description of the statistical parameters including central tendency (e.g. means) or other basic estimates (e.g. regression coefficient) AND variation (e.g. standard deviation) or associated estimates of uncertainty (e.g. confidence intervals)
☐	☒	For null hypothesis testing, the test statistic (e.g. F, t, r) with confidence intervals, effect sizes, degrees of freedom and P value noted<br>*Give P values as exact values whenever suitable.*
☒	☐	For Bayesian analysis, information on the choice of priors and Markov chain Monte Carlo settings
☒	☐	For hierarchical and complex designs, identification of the appropriate level for tests and full reporting of outcomes
☒	☐	Estimates of effect sizes (e.g. Cohen's d, Pearson's r), indicating how they were calculated

Our web collection on statistics for biologists contains articles on many of the points above.

Software and code

Policy information about availability of computer code

Data collection	EPU 2.21 software (Thermo Fisher Scientific)
Data analysis	cryoSPARC v3.2.0, Coot 0.8.9, Phenix 1.19.2-4158, Chimera 1.16, Pymol 2.5.1, HOLE 2.2.005, pCLAMP 10.7

For manuscripts utilizing custom algorithms or software that are central to the research but not yet described in published literature, software must be made available to editors and reviewers. We strongly encourage code deposition in a community repository (e.g. GitHub). See the Nature Portfolio guidelines for submitting code & software for further information.

Data

Policy information about availability of data

All manuscripts must include a data availability statement. This statement should provide the following information, where applicable:

- Accession codes, unique identifiers, or web links for publicly available datasets
- A description of any restrictions on data availability
- For clinical datasets or third party data, please ensure that the statement adheres to our policy

The coordinate files and cryo-EM maps of extended state of AtOSCA1.1, contracted state of AtOSCA3.1, extended state of AtOSCA3.1 and hTMEM63A in detergent have been deposited at Protein Data Bank (Electron Microscopy Data Bank (EMDB)) under accession codes PDBID-8GRN (EMD-34209), PDBID-8GRO (EMD-34210), PDBID-8GSO (EMD-34237) and PDBID-8GRS (EMD-34214), respectively. Structural models used to initiate model building were accessed from the Protein Data Bank under the accession codes 6JPF [https://www.rcsb.org/structure/6JPF].

Human research participants

Policy information about studies involving human research participants and Sex and Gender in Research.

Reporting on sex and gender	N/A
Population characteristics	N/A
Recruitment	N/A
Ethics oversight	N/A

Note that full information on the approval of the study protocol must also be provided in the manuscript.

Field-specific reporting

Please select the one below that is the best fit for your research. If you are not sure, read the appropriate sections before making your selection.

- [x] Life sciences
- [ ] Behavioural & social sciences
- [ ] Ecological, evolutionary & environmental sciences

For a reference copy of the document with all sections, see nature.com/documents/nr-reporting-summary-flat.pdf

Life sciences study design

All studies must disclose on these points even when the disclosure is negative.

Sample size	For electrophysiological recordings, each experiment was repeated at least eight times, and the results were reproduced each time.
Data exclusions	Cryo-EM micrographs with ice were excluded manually. Particles belonging to bad classes were discarded and the data processing flowchart were summarized in Supplementary Figs.
Replication	The frequency of electrophysiological replication exceeded 50%, while creating a typical giga-seal.
Randomization	All the successful electrophysiological recordings were allocated into experimental groups.
Blinding	The investigators were blinded to group allocation during data collection and analysis.

Reporting for specific materials, systems and methods

We require information from authors about some types of materials, experimental systems and methods used in many studies. Here, indicate whether each material, system or method listed is relevant to your study. If you are not sure if a list item applies to your research, read the appropriate section before selecting a response.

Materials & experimental systems

n/a	Involved in the study	
x		Antibodies
	x	Eukaryotic cell lines
x		Palaeontology and archaeology
x		Animals and other organisms
x		Clinical data
x		Dual use research of concern

Methods

n/a	Involved in the study	
x		ChIP-seq
x		Flow cytometry
x		MRI-based neuroimaging

Eukaryotic cell lines

Policy information about cell lines and Sex and Gender in Research

Cell line source(s)	Expi293F, sf9 and HEK293T cells were obtained from Thermo Fisher Scientific.
Authentication	Cell line was directly purchased from Distributors. Cell line authentication was not performed.
Mycoplasma contamination	Expi293F, sf9 and HEK293T cells were negative for mycoplasma contamination.

Commonly misidentified lines<br>(See ICLAC register)	No commonly misidentified cell lines used in this study.
